# Near-infrared spectroscopy parameters in patients undergoing continuous venovenous hemodiafiltration

**DOI:** 10.31744/einstein_journal/2019AO4439

**Published:** 2019-02-06

**Authors:** Renato Carneiro de Freitas Chaves, Philipe Franco do Amaral Tafner, Felipe Ko Chen, Letícia Bagatini Meneghini, Thiago Domingos Corrêa, Roberto Rabello, Miguel Cendoroglo, Oscar Fernando Pavão dos Santos, Ary Serpa

**Affiliations:** 1 Department of Critical Care Medicine, Hospital Israelita Albert Einstein, São Paulo, SP, Brazil.; 2 Laboratory for Critical Care Research, Hospital Israelita Albert Einstein, São Paulo, SP, Brazil.; 3 Department of Anesthesiology, Irmandade da Santa Casa de Misericórdia de Santos, Santos, SP, Brazil.; 4 Faculdade de Medicina do ABC, Santo André, SP, Brazil.; 5 Faculdade Israelita de Ciências da Saúde Albert Einstein, São Paulo, SP, Brazil.; 6 Department of Critical Care Medicine, Hospital Municipal Dr. Moysés Deutsch – M’Boi Mirim, São Paulo, SP, Brazil.; 7 Department of Nephrology, Hospital Israelita Albert Einstein, São Paulo, SP, Brazil.; 8 Department of Intensive Care, Academic Medical Center, Amsterdam, The Netherlands.

**Keywords:** Spectroscopy, near-infrared, Microcirculation, Oxygenation, Hemodynamics, Acute kidney injuries, Renal replacement therapy, Critical care, Espectroscopia de luz próxima ao infravermelho, Microcirculação, Oxigenação, Hemodinâmica, Lesão renal aguda, Terapia de substituição renal, Cuidados críticos

## Abstract

**Objective:**

To investigate the impacts of continuous venovenous hemodiafiltration on the microcirculation in patients with acute kidney injury.

**Methods:**

A prospective observational pilot study conducted in a 40-bed, open clinical-surgical intensive care unit of a private tertiary care hospital located in the city of São Paulo (SP), Brazil. Microcirculation was assessed using near-infrared spectroscopy by means of a 15mm probe placed over the thenar eminence. Vascular occlusion test was performed on the forearm to be submitted to near-infrared spectroscopy by inflation of a sphygmomanometer cuff to 30mmHg higher than the systolic arterial pressure. The primary endpoint was the assessment of near-infrared spectroscopy-derived parameters immediately before, 1, 4 and 24 hours after the initiation of continuous venovenous hemodiafiltration.

**Results:**

Nine patients were included in this pilot study over a period of 2 months. Minimum tissue oxygen saturation measured during the vascular occlusion test was the only near-infrared spectroscopy-derived parameter to differed over the time (decrease compared to baseline values up to 24 hours after initiation of continuous venovenous hemodiafiltration).

**Conclusion:**

The impacts of microcirculatory dysfunction on clinical outcomes of patients undergoing to continuous venovenous hemodiafiltration need to be further investigated.

## RESUMO

**Objetivo:**

Avaliar o impacto da hemodiafiltração venovenosa contínua na microcirculação de pacientes com lesão renal aguda.

**Métodos:**

Estudo piloto, prospectivo e observacional conduzido em uma unidade de terapia intensiva clínico-cirúrgica aberta, com 40 leitos, localizada em um hospital terciário, privado, na cidade de São Paulo (SP), Brasil. A microcirculação foi avaliada empregando-se a espectroscopia no infravermelho próximo, por meio de uma sonda de 15mm posicionada sobre a eminência tenar. O teste de oclusão vascular foi realizado no antebraço a ser submetido à espectroscopia no infravermelho próximo, inflando-se o manguito de um esfigmomanômetro a um valor 30mmHg acima da pressão arterial sistólica. O desfecho primário foi a avaliação dos parâmetros derivados por espectroscopia no infravermelho próximo imediatamente antes, 1, 4 e 24 horas após o início da hemodiafiltração venovenosa contínua.

**Resultados:**

Foram incluídos nove pacientes neste estudo piloto ao longo de 2 meses. A saturação de oxigênio tecidual mínima mensurada durante o teste de oclusão vascular foi o único parâmetro derivado por espectroscopia no infravermelho próximo que diferiu ao longo do tempo, com queda em relação aos valores iniciais nas primeiras 24 horas após o início da hemodiafiltração venovenosa contínua.

**Conclusão:**

A influência da disfunção microcirculatória sobre os desfechos clínicos de pacientes submetidos à hemodiafiltração venovenosa contínua precisa ser melhor investigada.

## INTRODUCTION

Acute kidney injury (AKI) is a frequent complication in critically ill patients, associated with increased morbidity and mortality.^(^ ^1^ ^)^ Renal replacement therapy (RRT) is sometimes needed to control azotemia, fluid balance and electrolyte and acid-base imbalances; it is a cornerstone of treatment in more severe cases.^(^ ^2^ ^)^ Although controversial, continuous RRT (CRRT) has become a routine therapy for AKI in many countries, given the lower hemodynamic compromise and higher renal recovery rates compared to intermittent hemodialysis.^(^ ^2^ ^)^

The impact of different therapeutic interventions on the microcirculation of critically ill patients has been reported.^(^ ^3^ ^)^ Microcirculatory dysfunction has also been associated with worse outcomes in septic shock patients^(^ ^4^ ^)^ and others.^(^ ^3^ ^)^ Microcirculatory dysfunction may translate into worse outcomes in patients submitted to RRT. In these patients, CRRT may be used to mitigate dysfunction due to higher hemodynamic stability and maintenance of sufficient perfusion pressure in the course of therapy.

Microcirculatory changes are known to play a role in pathophysiology of AKI. Still, only one study assessing microcirculatory changes associated with RRT in patients with AKI has been published to date.^(^ ^5^ ^)^

## OBJECTIVE

To address the impacts of continuous venovenous hemodiafiltration on the microcirculation of patients with acute kidney injury.

## METHODS

This study was conducted in compliance with STrengthening the Reporting of OBservational studies in Epidemiology (STROBE) guidelines.^(^ ^6^ ^)^

### Study design and setting

A prospective, observational pilot study conducted in a 40-bed, open clinical-surgical high-density intensive care unit (ICU) of a private tertiary care hospital located in the city of São Paulo (SP), Brazil. This study was approved by the Ethics Committee of *Hospital Israelita Albert Einstein*, protocol 1.467.160, CAAE: 54067916.4.0000.0071. Written Informed Consent was obtained from all participants or their next of kin.

### Inclusion and exclusion criteria

The following patients were consecutively included: (1) age ≥18 years; (2) affected with AKI (according to KDIGO criteria)^(^ ^7^ ^)^ and undergoing continuous venovenous hemodiafiltration (CVVHDF). Exclusion criteria were as follows: diagnosis of chronic kidney disease; pregnancy and high risk of death within the next 24 hours.

### Continuous venovenous hemodiafiltration protocol

Patients received CVVHDF according to pre-established local guidelines.^(^ ^8^ ^)^ In all cases, CVVHDF was indicated and conducted by expert nephrologists. Following CVVHDF indication, a triple lumen catheter (Arrow International, PA, USA) was inserted into the internal jugular or femoral vein.^(^ ^8^ ^)^ Continuous venovenous hemodiafiltration was performed using a Prisma machine (Gambro Renal Products, France).

The Prisma machine was primed with 1,000mL of normal saline solution.^(^ ^8^ ^)^ Blood samples were collected as dictated by the institutional protocol for determination of the optimal dialysis solution composition.^(^ ^8^ ^)^ Ionized calcium was measured every 6 hours. Sodium and potassium levels, and arterial blood gases were analyzed every 12 hours. Creatinine, urea, magnesium, total calcium, chloride and phosphorus levels were measured once daily.^(^ ^8^ ^)^

Standard dialysis solution consisted of sodium (110mEq/L), chloride (111mEq/L), magnesium (1.5mEq/L) and dextrose 0.1%.^(^ ^8^ ^)^ Sodium bicarbonate, potassium phosphate and magnesium sulfate were added as needed. Solution temperature was approximately 35°C.^(^ ^8^ ^)^

### Near-infrared spectroscopy monitoring

Following the indication of CVVHDF by the nephrologist, microcirculation was assessed using near-infrared spectroscopy (NIRS) (InSpectra Tissue Spectrometer model 650; Hutchinson Technology Inc., Hutchinson, MN, USA) by means of a 15mm probe placed over the thenar eminence.^(^ ^9^ ^)^ After a 3-minute period of minimal tissue oxygen saturation (SatO_2_) variation and NIRS signal stabilization, basal SatO_2_ was recorded and a vascular occlusion test (VOT) performed by inflating a sphygmomanometer cuff to 30mmHg higher than the systolic arterial pressure, as described elsewhere.^(^ ^9^ ^)^ The cuff was kept inflated for 3 minutes, then quickly deflated.^(^ ^9^ ^)^ SatO_2_ was continuously recorded for 5 minutes during the reperfusion phase. Near-infrared spectroscopy derived parameters were monitored immediately before CVVHDF initiation, then after 1, 4 and 24 hours.

Near-infrared spectroscopy derived parameters were calculated using a research software (Hutchinson Technology Inc., Hutchinson, MN, USA). SatO_2_ (%) and tissue hemoglobin index (THI) were measured at baseline. The descending slope (%/minute) was calculated from baseline to minimum SatO_2_ (SatO_2_min) immediately after the end of the VOT. The ascending slope (%/minute) was calculated from SatO_2_min to maximum SatO_2_ (SatO_2_max) immediately after the end of the VOT. The area under the curve of reactive hyperemia was calculated from SatO_2_max to return to baseline SatO_2_.

### Data collection and outcomes

Age, sex, body mass index (BMI), reason for ICU admission, comorbidities and the Simplified Acute Physiology Score (SAPS III) were recorded upon ICU admission. Systemic hemodynamic variables, ventilatory parameters, use of vasopressors and sedatives, capillary refill time, forearm-to-fingertip skin temperature gradient^(^ ^9^ ^)^ and the peripheral perfusion index (Radical-7 Pulse CO-Oximeter; Masimo Corporation, Irvine, CA, USA) were recorded at the time of NIRS measurement. Arterial blood gases were recorded as close as possible to NIRS measurement. The Sequential Organ Failure Assessment (SOFA) score was recorded during the first 24 hours following ICU admission. Outcomes of interest were length of in-hospital and ICU stay, ICU mortality, in-hospital mortality and 28-day mortality.

### Endpoints

The primary endpoint was the assessment of NIRS-derived parameters immediately before, then 1, 4, and 24 hours after CVVHDF initiation. The following parameters were assessed: baseline, minimum and maximum SatO_2_, descending and ascending slope, THI, recovery time and hyperemic area.^(^ ^9^ ^)^

### Statistical analysis

Data were reported as median (interquartile range) and absolute numbers (percentage). Parameters measured at the four experimental time points were analyzed using the univariate or the multivariate (adjusted for SOFA and SAPS III) linear mixed model, with patients introduced as random effect. The level of significance was set at p<0.05. Statistical analyses were performed using R 3.4.1 (R Foundation for Statistical Computing, Vienna, Austria).

## RESULTS

### Baseline characteristics

Nine patients were included over a period of 2 months. Most patients were men (77.8%), presented with sepsis-induced AKI (66.7%), and were admitted to ICU due to non-surgical reasons. Urinary output immediately before and 24 hours after the initiation of RRT was zero in all cases; 44.6% of patients required mechanical ventilation, and 33.3% received norepinephrine (Tables 1 and 2).

**Table 1:** Patient characteristics

Characteristics	(n=9)
Age, years	66 (61-76)
Male gender	7/9 (77.8)
BMI, kg/m^2^	26.20 (22.77-27.17)
Time from hospital admission to inclusion, days	3 (2-16)
Time from ICU admission to inclusion, days	2 (1-3)
Etiology of acute kidney injury	
Sepsis	6/9 (66.7)
Pancreatitis	1/9 (11.1)
Drug	1/9 (11.1)
Acute exacerbation of chronic renal disease	1/9 (11.1)
Type of ICU admission	
Medical	9/9 (100.0)
Surgical	0/9 (0.0)
Source of admission	
Emergency department	4/9 (44.4)
Step down unit	4/9 (44.4)
Other hospital	1/9 (11.1)
Comorbidities	
Hypertension	5/9 (55.5)
Active cancer	3/9 (33.3)
Congestive heart failure	2/9 (22.2)
Coronary insufficiency	1/9 (11.1)
*Diabetes mellitus*	1/9 (11.1)
Diagnosis upon admission	
Respiratory	3/9 (33.3)
Cardiovascular	1/9 (11.1)
Gastrointestinal	1/9 (11.1)
Metabolic	1/9 (11.1)
Neurological	1/9 (11.1)
Renal	1/9 (11.1)
Sepsis	1/9 (11.1)
SAPS III	60 (51-63)
SOFA	9 (5-11)
Baseline creatinine, mg/dL	1.00 (0.80-1.90)
Creatinine prior to RRT, mg/dL	2.83 (2.41-3.61)
Creatinine clearance prior to RRT, mL/minute	25.29 (11.94-37.10)
Urine output 24 hours prior to RRT, mL/kg/hour	0.63 (0.28-0.87)
Urea prior RRT, mg/dL	74 (58-87)
Arterial blood gas and vital signs	
pH	7.37 (7.25-7.42)
PaO_2_, mmHg	99.40 (93.85-145.50)
PaCO_2_, mmHg	39.10 (31.50-40.85)
Base excess, mEq/L	-3.05 (-13.08-2.90)
Lactate, mg/dL	12.00 (10.94-15.00)
Hemoglobin, g/dL	8.50 (8.10-11.10)
Mechanical ventilation	4/9 (44.4)
Respiratory rate, bpm	16 (15-19)
PaO_2_/FiO_2_	400 (311-457)
Tidal volume, mL/kg PBW	6.28 (5.84-9.11)
PEEP, cmH_2_O	10.0 (9.5-10.5)
CVVHDF characteristics	
Catheter in right internal jugular vein	8/9 (88.9)
Blood flow, mL/minute	100 (100-100)
Citrate flow, mL/hour	155 (150-160)
Dialysate flow, mL/hour	2,000 (2,000-2,000)
Replacement flow, mL/hour	600 (600-600)
Ultrafiltration rate, mL/hour	190 (150-250)
Dose, mL/kg/hour	37.10 (33.30-40.86)
Length of in-hospital stay (survivors), days	29 (23-59)
Length of ICU stay (survivors), days	12 (7-15)
ICU mortality	0/9 (0)
In-hospital mortality	2/9 (22.2)
28-day mortality	1/9 (11.1)

Data presented as median (interquartile range) or N/total (%). BMI: body mass index; ICU: intensive care unit; SAPS: Simplified Acute Physiology Score; SOFA: Sequential Organ Failure Assessment; PaO_2_: partial pressure of oxygen; PaCO_2_: partial pressure of carbon dioxide; FiO_2_: inspired oxygen fractions; PBW: predicted body weight; PEEP: positive end-expiratory pressure; CVVHDF: continuous venovenous hemodiafiltration.

**Table 2:** Vital signs and near-infrared spectroscopy-derived parameters during continuous venovenous hemodiafiltration

	Before CVVHDF	1 hour after CVVHDF	4 hours after CVVHDF	24 hours after CVVHDF	Unadjusted p value^*^	Adjusted p value^†^
Vital signs						
Heart rate, bpm	83 (73-113)	81 (76-104)	82 (69-88)	77 (65-85)	0.134	0.135
MAP, mmHg	83 (78-96)	80 (72-89)	83 (74-91)	87 (78-93)	0.458	0.415
CVP, mmHg	10 (8-11)	18 (6-19)	9 (8-11)	12 (10-12)	0.895	0.912
SpO_2_, %	97 (95-99)	97 (97-97)	97 (96-98)	97 (93-98)	0.487	0.478
Capillary refill time, second	1.86 (1.56-1.98)	2.20 (1.62-2.45)	2.57 (1.83-3.07)	2.47 (1.81-2.91)	0.070	0.078
Forearm-to-fingertip skin temperature gradient, °C	1.80 (- 0.10-3.40)	1.60 (0.10-1.90)	1.15 (0.35-2.93)	2.05 (0.80-2.83)	0.957	0.989
PPI, %	1.40 (0.72-2.00)	1.40 (0.73-1.60)	0.95 (0.53-1.78)	0.71 (0.56-1.00)	0.149	0.156
Norepinephrine						
Number of patients	3 / 9 (33.3)	3 / 9 (33.3)	3 / 9 (33.3)	3 / 9 (33.3)	---	---
μg/kg/minute	0.11 (0.07-0.52)	0.13 (0.07-0.66)	0.10 (0.06-0.65)	0.21 (0.15-0.37)	0.635	0.635
Urine output 24 hours after RRT, mL/kg/hour	---	---	---	0.08 (0.03-0.64)	---	---
Urine output 24 hours after RRT, mL/kg/hour	---	---	---	- 89 (- 1,070-1,083)	---	---
NIRS-derived parameters						
THI	11.8 (10.5-12.9)	9.0 (7.9-9.8)	9.6 (7. 2-11.4)	11.1 (7.6-11.9)	0.156	0.127
SatO_2_, %	83 (81-89)	80 (76-84)	81 (73-84)	81 (78-83)	0.227	0.187
SatO_2_ min, %	64 (49-67)	55 (54-60)	55 (51-59)	52 (43-59)	0.029	0.023
SatO_2_ max, %	94 (87-95)	88 (83-93)	90 (81-93)	90 (87-93)	0.237	0.210
Descending slope, %/minute	8.3 (4.4-10.4)	7.7 (6.0-9.8)	7.4 (7.3-8.6)	10.5 (10.4-12.5)	0.052	0.088
Ascending slope, %/second	1.6 (1.2-3.1)	2.2 (1.9-2.6)	1.9 (1.2-2.4)	2.7 (1.9-3.3)	0.656	0.607
Recovery time, second	31.0 (29.5-48.5)	34.0 (31.5-69.0)	28.5 (27.3-61.3)	23.0 (20.8-29.0)	0.264	0.435
SatO_2_max - SatO_2_min, %	7.0 (5.5-9.5)	8.0 (7.0-8.5)	6.0 (6.0-7.5)	9.0 (5.0-11.5)	0.943	0.956
Hyperemic area	11.0 (6.8-12.0)	11.5 (7.3-13.4)	9.4 (7.5-12.7)	13.9 (12.4-4.5)	0.474	0.500

Data presented as median (interquartile range) or n/total (%). * p values calculated using linear mixed models; ^†^ adjusted for Simplified Acute Physiology Score III e Sequential Organ Failure Assessment. CVVHDF: continuous venovenous hemodiafiltration; MAP: mean arterial pressure; CVP: central venous pressure; SpO_2_: pulse oximeter saturation; PPI: peripheral perfusion index; RRT: renal replacement therapy; NIRS: near-infrared spectroscopy; THI: tissue hemoglobin index; SatO_2_: tissue oxygen saturation; SatO_2_min: minimum tissue oxygen saturation after arterial occlusion test; StO_2_max: maximum tissue oxygen saturation after arterial occlusion.

### Continuous venovenous hemodiafiltration

Most patients received CVVHDF via a catheter placed in the right internal jugular vein (88.9%); blood, dialysis and replacement solution flow were kept constant. Median ultrafiltration rate and CVVHDF dose of corresponded to 190mL/hour and 37.1mL/kg/hour, respectively. All patients received citrate anticoagulation during CVVHDF.

### Vital signs and NIRS-derived parameters

Systemic hemodynamics and peripheral perfusion remained unchanged throughout the experimental period. Heart rate, mean arterial pressure, peripheral hemoglobin saturation as measured by pulse oximetry, forearm-to-fingertip skin temperature gradient and peripheral perfusion index did not change significantly during the first 24 hours of RRT. Minimum SatO_2_ was the only NIRS-derived parameter to differ during the VOT, with a decrease compared to baseline in the first 24 hours after CVVHDF initiation. This association remained significant after adjustment for baseline severity. Also, given hemoglobin levels and temperature may impact minimum SatO_2_ levels, further analysis adjusted for SOFA, SAPS III, baseline hemoglobin levels and temperature measured at different experimental time points was performed. Results of this analysis confirmed previous finding (p=0.017; SatO_2_ variation over time).

## DISCUSSION

In this pilot study, CVVHDF had no impact on clinically assessed macrocirculatory or microcirculatory parameters. Minimum SatO_2_ levels measured during VOT was the only NIRS-derived parameter to change (decrease over time) during CVVHDF.

Near-infrared spectroscopy does not allow direct measurement of blood flow and may therefore induce misinterpretation of SatO_2_ values.^(^ ^9^ ^,^ ^10^ ^)^ Analysis of SatO_2_ changes during a brief period of forearm ischemia VOT allows fast, dynamic assessment of the microvascular reserve.^(^ ^9^ ^,^ ^10^ ^)^ Consensus regarding VOT intensity and duration is lacking; however, the VOT used in this study has been well described in literature.^(^ ^9^ ^,^ ^10^ ^)^ Basically, the descending VOT slope is used to estimate thenar muscle oxygen consumption and the ascending slope and hyperemic area to estimate post-ischemic vasodilation and capillary recruitment.^(^ ^9^ ^,^ ^10^ ^)^

Deterioration of the microcirculation during the first 24 hours of CRRT has been reported.^(^ ^5^ ^)^ However, that study was based on a comparatively smaller number of variables. Drop in minimum SatO_2_ values during the experimental period was the only significant change in this study. Minimum SatO_2_ is thought to be an indicator of the extent of ischemia.^(^ ^11^ ^)^ This finding suggests higher oxygen consumption during the first 24 hours of CVVHDF. Also, changes detected in this study may have been caused by CVVHDF, as dialysis may induce pre-capillary sphincter constriction due to changes in electrolyte concentrations and drop in core temperature in response to dialysis against cool dialysate.^(^ ^5^ ^)^

The dissociation between macrocirculation and microcirculation has been described; however, macrocirculation parameters, such as heart rate, mean arterial pressure, cardiac output and central venous oxygen saturation are commonly used to monitor critically ill patients.^(^ ^10^ ^,^ ^12^ ^)^ Also, persistence of microcirculatory abnormalities in spite of macrocirculatory optimization is associated with higher mortality.^(^ ^4^ ^,^ ^10^ ^)^ Hence, microcirculatory monitoring, early optimization of microcirculatory parameters and accurate assessment of intravascular volume for appropriate intervention selection may translate into better outcomes in critically ill patients.^(^ ^10^ ^,^ ^13^ ^,^ ^14^ ^)^

This study has limitations. Being a pilot study with small sample size, it lacks sufficient power to confirm or exclude associations, which can only be suggested. Also, single-center design decreases the external validity of findings, particularly due to use of a specific protocol for CVVHDF in ICU. Moroever, NIRS does not measure microcirculatory flow directly and the NIRS signal is limited to vessels less than 1mm in diameter.^(^ ^10^ ^)^

## CONCLUSION

Minimum tissue oxygen saturation measured during the vascular occlusion test was the only near-infrared spectroscopy-derived parameter to change during the first 24 hours of continuous venovenous hemodiafiltration. Further studies with adequate sample size and power are warranted to address the impacts of microcirculatory dysfunction on clinical outcomes of patients undergoing to continuous venovenous hemodiafiltration.
